# LncRNAs Expression Signatures of Renal Clear Cell Carcinoma Revealed by Microarray

**DOI:** 10.1371/journal.pone.0042377

**Published:** 2012-08-06

**Authors:** Gan Yu, Weimin Yao, Ji Wang, Xin Ma, Wei Xiao, Heng Li, Ding Xia, Yang Yang, Kangli Deng, Haibing Xiao, Bohan Wang, Xiaolin Guo, Wei Guan, Zhiquan Hu, Yinqi Bai, Hua Xu, Jihong Liu, Xu Zhang, Zhangqun Ye

**Affiliations:** 1 Department of Urology, Tongji Hospital, Tongji Medical College, Huazhong University of Science and Technology, Wuhan, China; 2 Department of Urology and Helen-Diller Comprehensive Cancer Center, University of California San Francisco, San Francisco, California, United States of America; 3 Department of Urology, PLA General Hospital, Military Postgraduate Medical College, Beijing, China; 4 BGI-Shenzhen, Shenzhen, China; Baylor College of Medicine, United States of America

## Abstract

**Background:**

Long noncoding RNAs (lncRNAs) are an important class of pervasive genes involved in a variety of biological functions. They are aberrantly expressed in many types of cancers. In this study, we described lncRNAs profiles in 6 pairs of human renal clear cell carcinoma (RCCC) and the corresponding adjacent nontumorous tissues (NT) by microarray.

**Methodology/Principal Findings:**

With abundant and varied probes accounting 33,045 LncRNAs in our microarray, the number of lncRNAs that expressed at a certain level could be detected is 17157. From the data we found there were thousands of lncRNAs that differentially expressed (≥2 fold-change) in RCCC tissues compared with NT and 916 lncRNAs differentially expressed in five or more of six RCCC samples. Compared with NT, many lncRNAs were significantly up-regulated or down-regulated in RCCC. Our data showed that down-regulated lncRNAs were more common than up-regulated ones. ENST00000456816, X91348, BC029135, NR_024418 were evaluated by qPCR in sixty-three pairs of RCCC and NT samples. The four lncRNAs were aberrantly expressed in RCCC compared with matched histologically normal renal tissues.

**Conclusions/Significance:**

Our study is the first one to determine genome-wide lncRNAs expression patterns in RCCC by microarray. The results displayed that clusters of lncRNAs were aberrantly expressed in RCCC compared with NT samples, which revealed that lncRNAs differentially expressed in tumor tissues and normal tissues may exert a partial or key role in tumor development. Taken together, this study may provide potential targets for future treatment of RCCC and novel insights into cancer biology.

## Introduction

Incidence of renal cell carcinoma is the third highest in the urinary system tumors, accounting for approximately 3% of adult malignancies [1]. Kidney cancer is the sixteenth most frequent malignancies globally. 265731 individuals were diagnosed with renal caner and 113315 individuals died from this type of cancer in 2008 [2], [3]. More seriously, the morbidity and mortality of this type of cancer is still growing [4]. 90–95% of renal cell carcinoma arises from the kidney and the occurrence of tumors is associated with racial and ethnic characteristic [1].

In kidney tumors, RCCC is the most common type and the incidence of which is the highest in adult males in the past six decade. Basic research has shown that genetic events play a major role in the development of RCCC [5]. Loss of von Hipple-Lindau (VHL) from VHL gene mutation or aberrant chromatin remodeling and chromosomal loss at 3p lead to the deregulations of hypoxia inducible factor (HIF) family members which can directly or indirectly target some growth factor genes, which may be the most important genetic events in RCCC oncogenesis [6]–[8]. It has also been demonstrated that VHL mutation alone is insufficient to produce RCCC and additional genetic events are required [9]–[11]. The overwhelming majority transcriptional outputs of the mammalian genome were confirmed to be protein noncoding genes [12]. Those abundant transcriptomes were regarded as “transcriptional noise”. However, over the past decade, many studies have reported that those noncoding RNAs have series of important regulatory potential both in transcription and post transcription.

Noncoding RNAs are divided into short noncoding RNAs and long noncoding RNAs according to their length. MicroRNAs (miRs), the best studied short noncoding RNAs, have been showed different expression in numerous cancers and mir-34b, mir-29b, mir-210 and etc, have been confirmed to be involved in RCCC development [13]. LncRNAs are defined as noncoding RNAs that longer than 200 nucleotides in length. LncRNAs have been validated to have comprehensive function in biological processes through various of mechanisms [14]. More and more evidence indicated that lncRNAs exerted important roles in both normal development and diseases [12]. Misregulation of lncRNAs has been showed to be associated with many human diseases, including various types of cancers [15]. Large-scale analysis of full-length cDNA sequences have detected large numbers of long noncoding RNAs in human, mouse, and fly. These lncRNAs have been shown to exert key roles in imprinting control, cell differentiation, immune responses, human diseases, tumorigenesis and other biological processes [16]–[20]. But the expression of lncRNAs and their biological functions in RCCC still remain unknown.

In this study, we presented the lncRNAs expression profiles in six pairs of RCCC samples compared with NT samples, several of which were evaluated by qPCR in a total of sixty-three pairs of tissues. Our results demonstrated that lncRNAs expression profiles may provide new molecular biomarkers or a new basis for the diagnosis of RCCC.

## Results

### Overview of lncRNAs Profiles

From the lncRNAs expression profiles, differentially expressed lncRNAs can be found between RCCC and NT samples. The expression profiles of lncRNAs in paired samples were shown by calculating log fold change Tumor/Normal (T/N). Agreement formulated as follows: fold-change cut-off:2.0, fold-change, positive value indicates up-regulation and negative value indicates down-regulation. Log fold-change means log2 value of absolute fold-change. Fold-change and p value are calculated from the normalized expression. We determined thousands of differentially expressed human lncRNAs in RefSeq_NR, UCSC_knowngene, Ensembl, H-invDB, Fantom, Fantom_stringent, NRED, RNAdb, misc_lncRNA, UCR and lncRNA in six RCCC patients.

**Figure 1:**
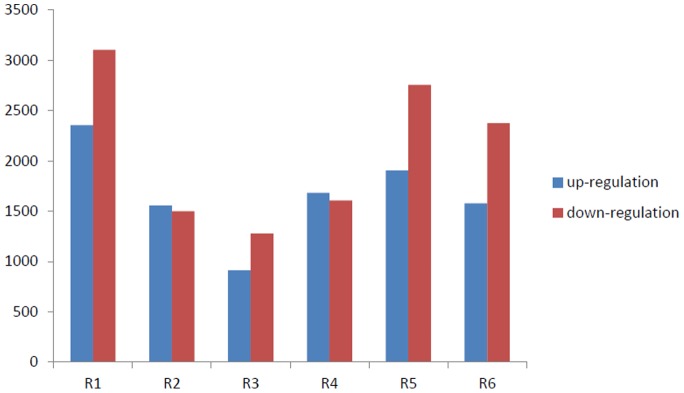
Counts of up-regulated and down-regulated lncRNAs. Many lncRNAs were determined to be significantly up-regulated or down-regulated in RCCC samples compared with NT samples in six patients by microarray. The counts of up-regulated and down-regulated lncRNAs varied across the six patients. In four out of six RCCC patients down-regulated lncRNAs were more common than up-regulated ones. Two patient down-regulated lncRNAs were less common than up-regulated ones.

Next, from the microarray data we compared the lncRNAs expression levels between the six RCCC tissues and their matched normal tissues, and identified an average of 3837 long lncRNAs (range from 2262–5532) that were significantly differentially expressed (≥2-fold) (Table S1). The basic information of the six patients was shown in Table S2. The sequence obtaining procedure may vary depending upon the source of the lncRNA. The results demonstrated that tens of thousands lncRNAs could be examined in normal and tumor tissues, but only thousands of lncRNAs were significantly up-regulated or down-regulated, which could be used to discriminate RCCC from matched normal tissues (Table S1, Table S3). Compared with the NT tissues, a total of 3055 lncRNAs were found to be consistently up-regulated or down-regulated shared by 4 of the 6 RCCC samples and 1866 by 5 and 626 in all (Table S4). ENST00000429695 (Log2 Fold change T/N = −4.175355) was the most significantly down-regulated one while ENST00000456816 (Log2 Fold change T/N = 6.312544) was the most significantly up-regulated (Table 1). The number of up-regulated and down-regulated lncRNAs varied in different patients. We found that down-regulated lncRNAs were more common than up-regulated ones (Figure 1).

**Table 1:** A collection of deregulated lncRNAs detected using microarray in six RCCC patients.

Down-regulated in cancer		Up-regulated in cancer	
lncRNAs	Log2 Fold change (T/N)	lncRNAs	Log2 Fold change (T/N)
ENST00000429695	−4.175355	ENST00000456816	6.312544
NR_024418	−4.164936	X91348	5.660148
uc004coq.3	−4.138873	uc009yby.1	4.520894
NR_024371	−4.017295	ENST00000514034	4.37416
ENST00000452496	−3.924731	ENST00000450687	3.567205
ENST00000435496	−3.684683	ENST00000412427	3.750126
ENST00000423174	−3.664962	uc002the.2	3.711211
ENST00000418694	−3.552973	NR_001458	3.650928
ENST00000439462	−3.482764	NR_026816	3.582491
BC029135	−3.440134	ENST00000460407	3.31089
ENST00000460134	−3.252417	ENST00000439042	2.990333
ENST00000421118	−3.241383	NR_027484	2.903099
BC031314	−3.208385	ENST00000458175	2.885053
ENST00000501056	−3.187289	ENST00000420287	2.871806
NR_024158	−3.021129	ENST00000376792	2.859402
ENST00000503579	−3.004994	BX647686	2.841756
uc002dhi.1	−2.826513	NR_024373	2.839526
ENST00000421424	−2.791417	BC012900	2.831941
ENST00000421314	−2.756638	NR_015421	2.830491
NR_023920	−2.722405	ENST00000445535	2.827482

RCCC samples versus NT samples, False discovery rate (FDR)<0.1%, p<0.01.

From the data of microarray, variations of lncRNAs and message RNAs expression was shown in Table 2. More and more evidence indicated that lncRNAs exerted important roles in gene expression. Thus, we explored the correlation between the expression of lncRNAs and the expression of protein coding genes. Four lncRNAs expression (ENST00000456816, X91348, BC029135 and NR_024418) were carried out using an absolute correlation coefficient cut off of greater than 0.85 and an FRD less than 0.01. We found that hundreds of genes were significantly associated with each of the four lncRNAs (Table S5).

**Table 2:** Summary of data from microarray for six pairs of renal cancer and adjacent normal tissues.

Table 2.1 Relative long noncoding RNA expression between six renal cancer and adjacent normal tissues.									
sample				Long noncoding RNA					
ID				Fold change 2–4	Fold change 4–6		Fold change>6	total	Changed lncRNAs
R1	Up-regulation			1979	242		134	2355	5460
	down-regulation			2400	589		116	3105	
R2	Up-regulation			1311	172		74	1557	3056
	down-regulation			1297	115		87	1499	
R3	Up-regulation			751	110		52	913	2190
	down-regulation			1163	67		47	1277	
R4	Up-regulation			1426	175		82	1683	3271
	down-regulation			1158	215		215	1588	
R5	Up-regulation			1607	176		120	1903	4659
	down-regulation			2482	233		41	2756	
R6	Up-regulation			1196	207		176	1479	3854
	down-regulation			2107	147		121	2375	
**Table 2** **.2 Relative message RNA expression between six renal cancer and adjacent normal tissues**									
**sample**			**Message RNA**						
			**Fold change 2**–**4**		**Fold change 4**–**6**	**Fold change>6**		**total**	**Changed mRNAs**
R1		Up-regulation	1669		279	241		2189	4329
		down-regulation	1657		340	143		2140	
R2		Up-regulation	1357		240	160		1757	3286
		down-regulation	1237		170	122		1529	
R3		Up-regulation	822		186	149		1157	2238
		down-regulation	862		98	121		1081	
R4		Up-regulation	1204		208	179		1591	3565
		down-regulation	1309		332	333		1974	
R5		Up-regulation	1636		315	271		2222	4085
		down-regulation	1660		130	73		1863	
R6		Up-regulation	1393		265	219		1877	3750
		down-regulation	1535		141	197		1873	

### LncRNA Classification and Subgroup Analysis

Recently, some classes or clusters of lncRNAs such as HOX lncRNAs and the lncRNAs with enhancer-like function (LncRNA-a), have been identified as specific function in human cells [21], [22]. Those subgroup lncRNAs are more likely to involve in the occurrence and development of many diseases, especially that of cancers. Numerous lncRNAs were found to be transcribed from within the human homeobox transcription factors (HOX) clusters [22]. Those lncRNAs expressed in a temporal and site-specific fashions probably used the same enhancers as the HOX genes and may have global regulating functions as the HOX. In current study, the profiling data of all probes targeting 407 discrete transcribed regions in the four human HOX loci for both LncRNAs and coding transcripts was presented in the Table S6. These data suggested that 148 coding transcripts could be detected in RCCC with 71 of them differentially expressed compare with the adjacent normal tissues. Then, about 257 lncRNAs transcribed were detected and 142 of them were found differently expressed in human HOX loci in RCCC. Profiling data of all probes for lncRNAs based on John Rinn’s papers was also provided in Table S7 and 751 lncRNAs within 1787 lncRNAs detected were differently expressed [23], [24].

Harrow and his colleagues have defined a set of lncRNAs in human cell lines as lncRNAs with enhancer-like function [21]. Depletion of those lncRNAs led to decreased expression of their neighboring protein-coding genes. The Table S8 contains profiling data of all probes for LncRNAs with enhancer-like function and 846 lncRNAs were detected while 357 of them differentially expressed. Differentially expressed enhancer-like LncRNAs and their nearby coding genes (distance<300 kb) were showed in Table S9. Previous studies indicated that lncRNAs have intrinsic cis-regulatory capacity because it can function while remaining tethered to its own locus. So we also provided differentially expressed lncRNAs and nearby coding gene pairs (distance<300 kb) for each comparison between samples. Pairs range from 113 to 344 for the six samples pairs was presented in Table S10 (p<0.05).

### Overview of mRNAs Profiles

Up to 13,664 coding transcripts could be detected in six pairs of samples through 30,215 coding transcripts probes (Table S11). Among the six pairs of samples, an average of 1899 mRNAs (range from 1591–2222) were up-regulated in RCCC compared with the matched normal tissues, while an average of 1743 mRNAs (range from 1081–2140) down-regulated (Table S12). GO and Pathway analysis showed that the different expressed mRNAs might involve in some of primary substance metabolism, and oxidation and peroxidization associated signal pathway. These results support the viewpoint that kidney cancer is a metabolic disease.

### Real-time Quantitative PCR Validation

**Figure 2:**
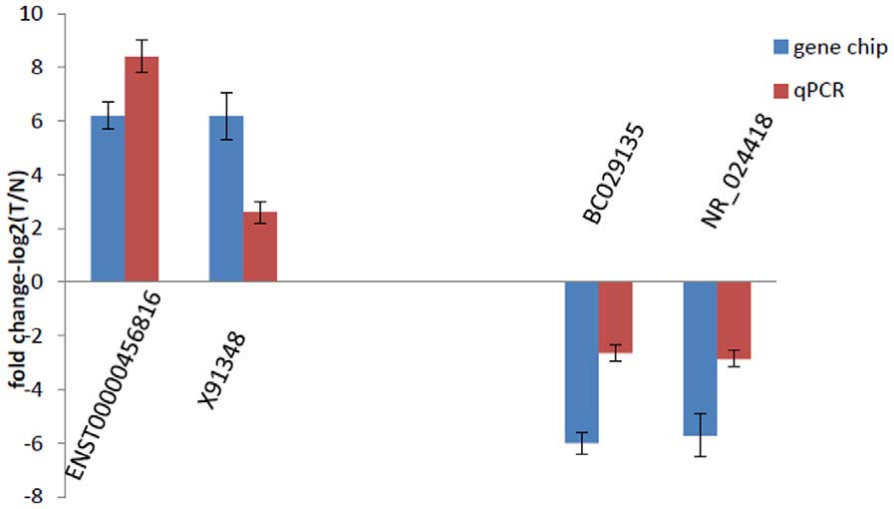
Comparison between microarray data and qPCR result. ENST00000456816, X91348, BC029135 and NR_024418 determined to be differentially expressed in RCCC samples compared with NT samples in six patients by microarray were validated by qPCR. The heights of the columns in the chart represent the log-transformed median fold changes (T/N) in expression across the six patients for each of the four lncRNAs validated; the bars represent standard errors. The validation results of the four lncRNAs indicated that the microarray data correlated well with the qPCR results.

In the first place, we examined the expression of these four lncRNAs (ENST00000456816, X91348, BC029135 and NR_024418) in two sets renal tissues using qPCR (Table S13). These data supported a strong consistency between the qPCR result and microarray data (Figure 2). Additionally, these four lncRNAs expression have also been analyzed in the five RCCC cell lines using qPCR (data not shown). The results demonstrated that ENST00000456816, X91348 were up-regulated and BC029135, NR_024418 were down-regulated in RCCC samples compared with NT samples. (p<0.001 for each lncRNAs) (Table S14).

### Construction of the Coding-non-coding Gene Co-expression Network

A coding-non-coding gene co-expression network (CNC network) was constructed based on the correlation analysis between the differential expressed lncRNA and mRNA. LncRNAs and mRNAs with Pearson correlation coefficients not less than 0.99 were selected to draw the network by the program of cytoscape. Among this co-expression network, 323 lncRNAs and 196 mRNAs composed the CNC network node. 519 network nodes made associated 1039 network pairs of co-expression lncRNAs and mRNAs, and most of pairs presented as positive correlation. The CNC network indicated that one mRNA could correlate with one to tens of lncRNAs and so were the lncRNAs. The CNC network presented in Table S15 might implicate the inter-regulation of lncRNAs and mRNAs in RCCC.

## Discussion

Previously, there are no reports describing lncRNAs expression profiles in RCCC, and there are no studies on the association of lncRNAs expression with clinical characteristics and outcomes in RCCC. RCCC is a genetic disease like other cancers which are caused by a series of genetic alterations [5]. Description of thousands of genomic sequences along with the technological development to identify the gene expression profiles on a large scale has provided a remarkable improvement in the analysis of carcinogenesis [25]. The improved technology may help us finding of possible therapeutic targets and identification of new diagnostic and prognostic markers. Therefore, deep research on genetic characterization will help to understand the occurrence and development of cancer, and facilitate the development of new personalized therapeutic strategies. Some studies have shown that lncRNAs expression can be de-regulated in most human cancers [26]–[29]. Some of the previous studies have shown that lncRNAs like HOTAIR played an important role in the development and progression of tumors [26].

This study focused on description of the lncRNAs expression profiles of RCCC, and explanation of differences between the tumor tissues compared with negative control (adjacent normal renal tissues). We analyzed 6 pairs of RCCC and adjacent NT samples by microarray and selected limited lncRNAs for validation by qPCR in sixty-three pairs of samples. With abundant and varied probes accounting 33,045 lncRNAs and 30,215 coding transcripts in the microarray, a large number of lncRNAs can be quantitatively determined. Comprehensive in-depth analysis of the expression profiles of lncRNAs in this carcinoma was executed in order to understand the role of lncRNAs in RCCC development and progression. Based on the microarray data, we found that tens of thousands of lncRNAs were expressed while thousands of which were differently expressed in each sample. 146 lncRNAs were identified up-regulated and 480 down-regulated in all 6 samples and most of which have not been functionally characterized. Those lncRNAs may involve in the development and progression of RCCC and it may provide novel path for better understanding of the molecular basis of RCCC.

Based on the previous work and computer analysis, four lncRNAs (ENST00000456816, X91348, BC029135 and NR_024418) were selected to validate the consistency. Expressions of these four lncRNAs were further evaluated by qPCR through sixty-three pairs of samples. LncRNA ENST00000456816 is a 796 bp intragenic lncRNA transcripted from the gene ENSG00000214145 (RP11–513G11.1) located on Chromosome 3q. This gene has other 3 transcripts all of which could not been detected among our samples. Some genes, such as PBRM1 located on chromosome 3p, in relative close vicinity to the VHL gene which is the vast majority mutation of RCCC play important roles in RCCC [5]. Though the lncRNA located on 3q is far from the VHL gene or PBRM1 gene, it can not exclude the possibilities that the lncRNA can recruit chromatin-modifying enzymes to target these two genes in trans. Whether this lncRNAs could play a role in RCCC needs further study. X91348 is a 1284 bp intragenic lncRNA transcripted from DiGeorge syndrome critical region gene 5 (DGCR5). This gene located on Chromosome 22 produces other 6 noncoding transcripts but no coding product. The novel transcript disrupted by a balanced translocation associated with DiGeorge syndrome was identified in 1996 by Sutherland, H, et al [30]. But the biological function of this RNA still remains elusive. BC029135 is a 723 bp intragenic lncRNA transcribed from chr10 and NR_024418 is a 723 bp intragenic lncRNA transcribed from chr5. Both of the two RNAs were uncharacterized and the biological functions of them were still unclear.

**Figure 3:**
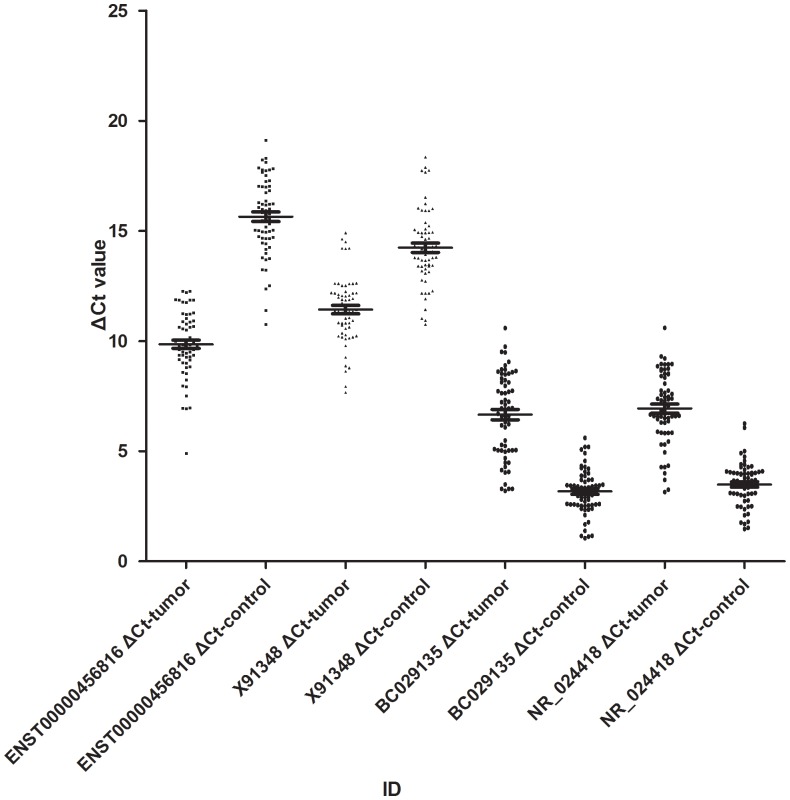
Distributions of the lncRNAs expression levels. That most significantly different expressed were validated by qPCR in 63 pairs of RCCC and NT samples.

The qPCR results matched well with the data of microarray and revealed that there was variability of lncRNAs expression in these tissues (Figure 3). It was likely to provide potential way to distinguish tumor tissues and adjacent normal tissues. Although noncoding RNAs in body fluid, such as serum, urine and etc, had been identified as possible biomarkers [31], it was too early for us to utilize the four lncRNAs as the possible biomarkers in RCCC just based on the present data which will be beneficial to explore novel molecular markers in RCCC. We also found that many lncRNAs expressions were significantly correlated with hundreds of protein coding genes expressions. In theory, lncRNAs have intrinsic cis-regulatory capacity and it has been confirmed, on the other hand the cis-regulatory mechanisms of lncRNAs have been described. More and more evidences confirmed that lncRNAs can act in both cis and trans [21], [32], [33] and the precise proportion of cis regulators requires more direct experimental approaches. Recently, it have been reported that more than a thousand lncRNAs are evolutionarily conserved in mammalian genomes and thus presumably function in diverse biological processes. Some studies have demonstrated that lncRNAs are involved in transcriptional regulation and its expression can be deregulated in human cancers [26]–[29]. Another team of researchers identified a new class of lncRNAs with an enhancer-like function in various human cell lines [21]. Knockdown or low expression of these lncRNAs led to decreased expression of their neighboring protein-coding genes, including several master regulators of cellular differentiation. Our microarray displayed a portion of enhancer-like lncRNAs (Table S8). Like classical enhancers, lncRNAs are orientation independent and require a minimal promoter in their target genes to enhance their transcriptions. Although the precise molecular mechanism is yet to be defined, this group of lncRNAs illustrates that eukaryotic transcription is very tightly regulated by overlapping mechanisms. All of the examples described indicate that lncRNAs can serve as a portion markers of active regulatory pathways [14]. More about this, the lncRNAs researched here should be distinguished from transcripts that are produced at enhancer sites [34], [35], the function of which has yet to be determined.

We also identified the differentially expressed lncRNAs and nearby coding gene pairs (Table S10). It was reported that knockdown or low expression of certain lncRNAs can led to decreased expression of their neighboring protein-coding genes, including several master regulators of cellular differentiation and that lncRNAs and nearby coding genes may represent shared upstream regulation or local transcriptional effects [24], [36]–[39]. Thus, the subgroup analysis of lncRNAs may help us to explore the relationship between lncRNAs and RCCC. Most of the lncRNAs have a distinct spatial and temporal specificity in the process of organismal differentiation and development. One study for 1300 lncRNAs of mice illustrated that in different parts of the brain tissue, lncRNAs have different expression patterns [40] and lncRNAs expression signatures have been described in prostate carcinoma, hepatic tumor [41]. Then in the development of RCCC, there may be different expression patterns of lncRNAs and the differentially expressed lncRNAs may execute special cellular function in RCCC. One previous study have shown the antitumor DNA topoisomerase I (Top1) inhibitor camptothecin (CPT) increases the cellular levels of two antisense lncRNAs at the 5′ (5′aHIF-1α) and 3′ (3′aHIF-1α) ends of the human HIF-1α gene, they also proved the two antisense lncRNAs at the 5′ (5′aHIF-1α) and 3′ (3′aHIF-1α) that induced nuclear membrane trafficking and response to partially different kinds of stress expressed in human kidney cancer tissues [42].

To the best of our knowledge, this is the first study that describes the expression profiles of human lncRNAs in RCCC by microarray, a collection of deregulated lncRNAs were aberrantly expressed in RCCC compared to matched NT sample groups. Probably, these deregulated lncRNAs play a key or partial role as oncogenes or tumor suppressors in the development and/or progression of this carcinoma. More work will be needed to determine whether these lncRNAs can serve as new therapeutic targets and diagnostic biomarkers in RCCC.

## Materials and Methods

### Patient Samples

Written informed consent was obtained from all patients and the study was approved by the Institutional Review Board of Huazhong University of Science and Technology, Tongji Medical College, Tongji Hospital. Sixty-three patients with clear cell carcinoma of kidney who received nephrectomy or partial nephrectomy were included in the study. Of these patients, six were used for microarray analysis of lncRNAs and fifty-seven were used for an extra evaluation. Clear cell carcinoma of kidney was diagnosed histopathologically. Clear cell carcinoma of kidney and matched histologically normal renal tissue from each subject were snap-frozen in liquid nitrogen immediately after resection. Detailed information of six clear cell carcinoma patients in microarray set is summarized in Table S2.

### RNA Extraction

The proportion of cancer cells in a tissue section was 100%, the frozen block was subjected to RNA extraction. Total RNA was extracted from sixty-three pairs of snap frozen clear cell carcinoma and matched histologically normal renal tissues using TRIzol reagent (Invitrogen, Carlsbad, CA, USA) according to the manufacturer’s protocol. The RNA integrity was evaluated by Nano Drop ND-1000 spectrophotometer.

### Microarray and Computational Analysis

RNA purified from total RNA after removal of rRNA was amplified and transcribed into fluorescent cRNA along the entire length of the transcripts without 3′ bias utilizing a random priming method and cDNA was labeled and hybridized to the Human LncRNA Array v2.0 (8×60 K, Arraystar). 33,045 LncRNAs and 30,215 coding transcripts which collected from the most authoritative databases such as RefSeq, UCSC Knowngenes, Ensembl and many related literatures can be detected by the microarray. Arraystar LncRNA Array Protocol: Step 1, Prepare the RNA Sample, kit and reagents: TRIzol® Reagent (Invitrogen life technologies), Biopulverizer (biospec), and Mini-Bead-Beater-16 (biospec). Step 2, Total RNA Clean-up and RNA QC. Step 3, Prepare labeling reaction. Step 4, Purify the labeled/amplified RNA and labeled Crna QC. Step 5, Hybridization. Step 6, Microarray Wash. Step 7, Scanning. Step 8, Extract data using Agilent Feature Extraction Software. More details would be shown in Table S18. The arrays were scanned by the Agilent Scanner G2505B and the acquired array images were analyzed by Agilent Feature Extraction software (version 10.7.3.1). Quantile normalization and subsequent data processing were performed using the GeneSpring GX v11.5.1 software package (Agilent Technologies). The microarray work was performed by KangChen Bio-tech, Shanghai P.R. China.

### Construction of the Coding-non-coding Gene Co-expression Network

The network construction procedures include that: (i) preprocess data: the same coding gene with different transcripts take the median value represents as the gene expression values, without special treatment of lncRNA expression value; (ii) screen data: remove the subset of data according to the lists that shown the differential expression of lncRNA and mRNA; (iii) calculate the Pearson correlation coefficient and use R value to calculate the correlation coefficient of PCC between lncRNA coding genes; and (iv) screen by Pearson correlation coefficient, selected the part that PCC≥0.99 as meaningful and draw the NCN network by cytoscape.

Pink node represents as the up-regulated lncRNA, blue node represents as the down-regulated lncRNA; Red node represents as the up-regulated mRNA, the green node represents as the down-regulated mRNA. Circular node represents as mRNA, parallelogram node represents as the lncRNA. Solid lines indicate a positive correlation; the dashed line indicates a negative correlation. The detail information was presented in Table S15.

### Q-PCR and Statistical Methods

Total RNA was extracted from frozen tumor specimens using TRIzol reagent (Invitrogen Life Technologies) and then reverse-transcribed using Fermentas RT reagent Kit (Perfect Real Time) according to the manufacturer’s instructions. LncRNAs expression in renal cancer tissues was measured by qPCR using SYBR Premix Ex Taq on MX3000 instrument. The primers used in this study were shown in (Table S16). Four lncRNAs that significantly deregulated (ENST00000456816, X91348, BC029135 and NR_024418) were evaluated in all of the patients included in this study. 2 µg of total RNA was converted to cDNA according to the manufacturer’s protocol. PCR was performed in a total reaction volume of 25 µl, including 10 µl SYBR Premix Ex Taq (2x), 1 µl of PCR Forward Primer (10 µM), 1 µl of PCR Reverse Primer (10 µM), 0.5 µl ROX Reference Dye II (50x)*3, 2 µl of cDNA, 8 µl of double-distilled water. The quantitative real-time PCR reaction was set at an initial denaturation step of 10 min at 95°C; and 95°C (5 seconds), 63°C (30 seconds), 72°C (30 seconds) in a total 40 cycles with a final extension step at 72°C for 5 min. All experiments were done in triplicate. All samples normalized to GAPDH. The median in each triplicate was used to calculate relative lncRNAs concentrations (ΔCt = Ct median lncRNAs-Ct median GAPDH). Expression fold changes were calculated using 2-ΔΔCt methods [41]. The lncRNAs expression differences between cancer and control were analyzed using Student’s t test within SPSS (Version 16.0 SPSS Inc.). A value of p<0.05 was considered as statistically significant.

## Supporting Information

Table S1 Differentially Expressed LncRNAs.
(XLS)

Table S2 Basic Medical Records of six Patients.
(XLSX)

Table S3 LncRNAs Expression Profiling Data.
(XLS)

Table S4 The Number of each Differentially Expressed LncRNA in six pairs of Samples.
(XLS)

Table S5 Correlation analysis of four lncRNAs.
(XLS)

Table S6 HOX cluster Profiling.
(XLS)

Table S7 Rinn lincRNAs profiling.
(XLS)

Table S8 Enhancer LncRNAs Profiling.
(XLS)

Table S9 Enhancer LncRNAs nearby coding gene data table.
(XLS)

Table S10 LncRNAs nearby Coding gene data table.
(XLS)

Table S11 mRNAs Expression Profiling Data.
(XLS)

Table S12 Differentially Expressed mRNAs.
(XLS)

Table S13 Delta-Ct values of Real-Time qPCR in sixty-three RCCC Patients and p value.
(XLSX)

Table S14 Delta-Ct values of Real-Time qPCR in sixty-three RCCC Patients.
(DOC)

Table S15 CNC network.
(PDF)

Table S16 Primer Catalog.
(XLSX)
