# Supplementary material for: LncRNAs Expression Signatures of Renal Clear Cell Carcinoma Revealed by Microarray
Source: PLoS One. 2012 Aug 6;7(8):e42377. doi: 10.1371/journal.pone.0042377 (PMC3412851; doi:10.1371/journal.pone.0042377)
Supplement: Table S14 — Delta-Ct values of Real-Time qPCR in sixty-three RCCC Patients. (DOC) [file pone.0042377.s014.doc]

**Table S3 Delta-Ct values of Real-Time qPCR in sixty-six renal clear cell carcinoma patients**

| Case No. | ENST00000456816 | | X91348 | | BC029135 | | NR_024418 | |
| --- | --- | --- | --- | --- | --- | --- | --- | --- |
|  | ΔCt-tumor | ΔCt-control | ΔCt-tumor | ΔCt-control | ΔCt-tumor | ΔCt-control | ΔCt-tumor | ΔCt-control |
| R39 | 9.46 | 16.84 | 12.25 | 14.19 | 8.16 | 2.62 | 7.05 | 3.29 |
| R64 | 10.88 | 17.75 | 10.63 | 13.41 | 6.98 | 3.37 | 7.54 | 3.45 |
| R72 | 6.93 | 17.26 | 10.1 | 14.32 | 4.51 | 4.24 | 5.8 | 4.04 |
| R74 | 9.01 | 18.12 | 12.06 | 13.39 | 6.12 | 4.95 | 5.82 | 3.98 |
| R47 | 10.56 | 10.76 | 10.82 | 10.75 | 6.62 | 3.47 | 7.73 | 4.37 |
| R46 | 8.58 | 15.48 | 8.78 | 13.18 | 3.23 | 1.18 | 3.12 | 1.5 |
| R44 | 12.21 | 16 | 11.88 | 12.17 | 4.07 | 2.38 | 7.33 | 3.42 |
| R32 | 9.15 | 15.34 | 10.81 | 13.79 | 8.65 | 2.55 | 8.5 | 2.99 |
| R31 | 9.84 | 16.19 | 11.33 | 14.91 | 8.52 | 4.1 | 8.93 | 3.64 |
| R33 | 9.3 | 14.99 | 10.23 | 13.76 | 7.76 | 3.19 | 8.49 | 3.59 |
| R34 | 11.78 | 14.71 | 12.6 | 13.41 | 5.07 | 2.59 | 6.55 | 3.04 |
| R89 | 9.78 | 13.8 | 14.2 | 17.75 | 6.73 | 3.43 | 6.43 | 2.46 |
| R27 | 10.17 | 17.29 | 11.63 | 13.92 | 8.62 | 3.47 | 8.05 | 4.29 |
| R38 | 7.52 | 17.52 | 10.72 | 13.78 | 6.93 | 3.73 | 7.58 | 3.54 |
| R40 | 9.63 | 13.76 | 11.62 | 13.08 | 4.51 | 4.03 | 5.81 | 4.14 |
| R42 | 11.21 | 15.04 | 12.07 | 13.48 | 6.21 | 4.59 | 5.28 | 3.96 |
| R45 | 9.65 | 15.87 | 10.83 | 10.93 | 6.62 | 3.74 | 7.73 | 4.73 |
| R52 | 9.84 | 16.23 | 8.87 | 13.81 | 3.32 | 1.81 | 8.39 | 3.76 |
| R51 | 9.16 | 15.98 | 11.93 | 12.71 | 4.72 | 2.83 | 6.57 | 3.08 |
| R53 | 12.26 | 17.01 | 10.18 | 13.69 | 8.56 | 2.54 | 8.69 | 3.76 |
| R54 | 11.87 | 14.17 | 12.17 | 14.67 | 8.26 | 4.32 | 6.48 | 2.72 |
| R55 | 11.88 | 17.87 | 11.34 | 15.92 | 7.67 | 3.92 | 8.64 | 3.92 |
| R56 | 10.01 | 19.12 | 12.61 | 14.04 | 5.08 | 2.95 | 9.28 | 6.23 |
| R57 | 9.98 | 16.08 | 11.98 | 14.29 | 6.34 | 3.47 | 8.73 | 6.04 |
| R58 | 9.65 | 17.84 | 14.21 | 17.67 | 8.34 | 3.64 | 3.23 | 1.67 |
| R59 | 10.63 | 16.74 | 12.61 | 14.32 | 7.28 | 3.43 | 3.68 | 1.77 |
| R60 | 7.93 | 16.23 | 10.22 | 13.65 | 5.32 | 2.37 | 4.26 | 2.07 |
| R61 | 6.98 | 17.02 | 10.81 | 12.76 | 8.56 | 3.48 | 6.67 | 4.02 |
| R62 | 9.86 | 15.19 | 11.87 | 12.16 | 5.06 | 2.63 | 7.26 | 4.38 |
| R63 | 11.76 | 15.71 | 11.46 | 12.17 | 4.18 | 2.83 | 6.27 | 3.42 |
| R77 | 11.23 | 17.03 | 10.13 | 14.26 | 6.48 | 4.27 | 6.56 | 3.05 |
| R65 | 10.04 | 18.23 | 10.93 | 13.67 | 7.73 | 3.91 | 7.29 | 4.06 |
| R66 | 9.63 | 17.03 | 12.52 | 14.18 | 8.93 | 2.74 | 7.05 | 3.43 |
| R68 | 10.89 | 17.78 | 10.36 | 13.14 | 7.26 | 3.73 | 7.45 | 3.54 |
| R73 | 6.95 | 18.31 | 10.18 | 14.26 | 8.17 | 2.64 | 5.82 | 4.07 |
| R78 | 9.02 | 17.67 | 10.28 | 11.02 | 6.99 | 3.38 | 5.28 | 3.99 |
| R79 | 12.26 | 16.37 | 11.86 | 12.27 | 6.64 | 3.49 | 7.36 | 3.45 |
| R81 | 9.51 | 15.43 | 11.34 | 14.92 | 4.09 | 2.42 | 8.94 | 3.67 |
| R82 | 11.87 | 14.76 | 12.62 | 13.43 | 8.67 | 2.59 | 6.56 | 3.08 |
| R85 | 9.67 | 14.98 | 11.36 | 14.94 | 7.78 | 3.24 | 8.94 | 3.95 |
| R87 | 11.26 | 16.24 | 12.63 | 13.46 | 5.06 | 2.62 | 7.63 | 3.64 |
| R88 | 11.04 | 15.04 | 12.52 | 14.91 | 9.52 | 5.11 | 5.42 | 3.63 |
| R97 | 10.92 | 14.68 | 14.21 | 17.74 | 3.32 | 1.19 | 6.34 | 2.74 |
| R 98 | 10.78 | 13.71 | 14.63 | 17.89 | 9.54 | 5.23 | 6.57 | 3.06 |
| R 103 | 9.37 | 14.26 | 11.26 | 14.04 | 9.78 | 5.64 | 7.43 | 4.26 |
| R 107 | 9.73 | 14.66 | 12.17 | 15.06 | 4.32 | 2.14 | 8.7 | 2.34 |
| R 108 | 8.85 | 15.84 | 12.19 | 15.08 | 8.74 | 3.51 | 6.49 | 2.48 |
| R 109 | 9.37 | 15.02 | 11.72 | 14.74 | 7.67 | 2.43 | 7.37 | 3.92 |
| R 110 | 11.87 | 15.79 | 11.06 | 14.07 | 5.07 | 2.94 | 6.82 | 4.98 |
| R 111 | 10.63 | 14.67 | 12.04 | 16.04 | 3.32 | 1.16 | 6.28 | 4.89 |
| R 112 | 11.29 | 15.93 | 12.18 | 15.94 | 6.26 | 4.37 | 4.32 | 2.13 |
| R102 | 10.67 | 14.96 | 10.57 | 14.62 | 6.97 | 3.39 | 5.86 | 4.26 |
| R104 | 9.36 | 13.99 | 11.42 | 15.37 | 8.75 | 2.93 | 8.47 | 2.96 |
| R105 | 9.84 | 16.29 | 11.29 | 14.93 | 7.36 | 3.04 | 8.92 | 3.97 |
| R106 | 9.51 | 14.43 | 12.11 | 16.24 | 5.01 | 2.62 | 6.59 | 3.04 |
| R91 | 8.54 | 15.56 | 7.94 | 13.27 | 10.62 | 3.21 | 10.58 | 3.99 |
| R87 | 8.8 | 13.23 | 9.79 | 15.23 | 5.13 | 3.01 | 4.93 | 3.99 |
| R83 | 9.69 | 12.53 | 9.26 | 11.91 | 8 | 5.23 | 8.84 | 4.54 |
| R37 | 9.27 | 13.25 | 11.03 | 16.01 | 9.09 | 3.18 | 9.19 | 3.33 |
| R30 | 4.9 | 11.39 | 8.63 | 16.53 | 7.15 | 2.57 | 8.32 | 1.45 |
| R27 | 8.25 | 14.47 | 14.91 | 15.92 | 3.52 | 1.71 | 4.26 | 2.47 |
| R25 | 7.97 | 12.38 | 7.67 | 11.43 | 5.27 | 1.42 | 3.98 | 1.74 |
| R21 | 10.51 | 17.78 | 14.5 | 18.35 | 5.52 | 1.08 | 6.64 | 2.45 |
| Note: p<0.0001 for each lncRNA, Student’s *t* test | | | | | | | | |
