# Supplementary figures and images for: LncRNAs Expression Signatures of Renal Clear Cell Carcinoma Revealed by Microarray
Source: PLoS One. 2012 Aug 6;7(8):e42377. doi: 10.1371/journal.pone.0042377 (PMC3412851; doi:10.1371/journal.pone.0042377)

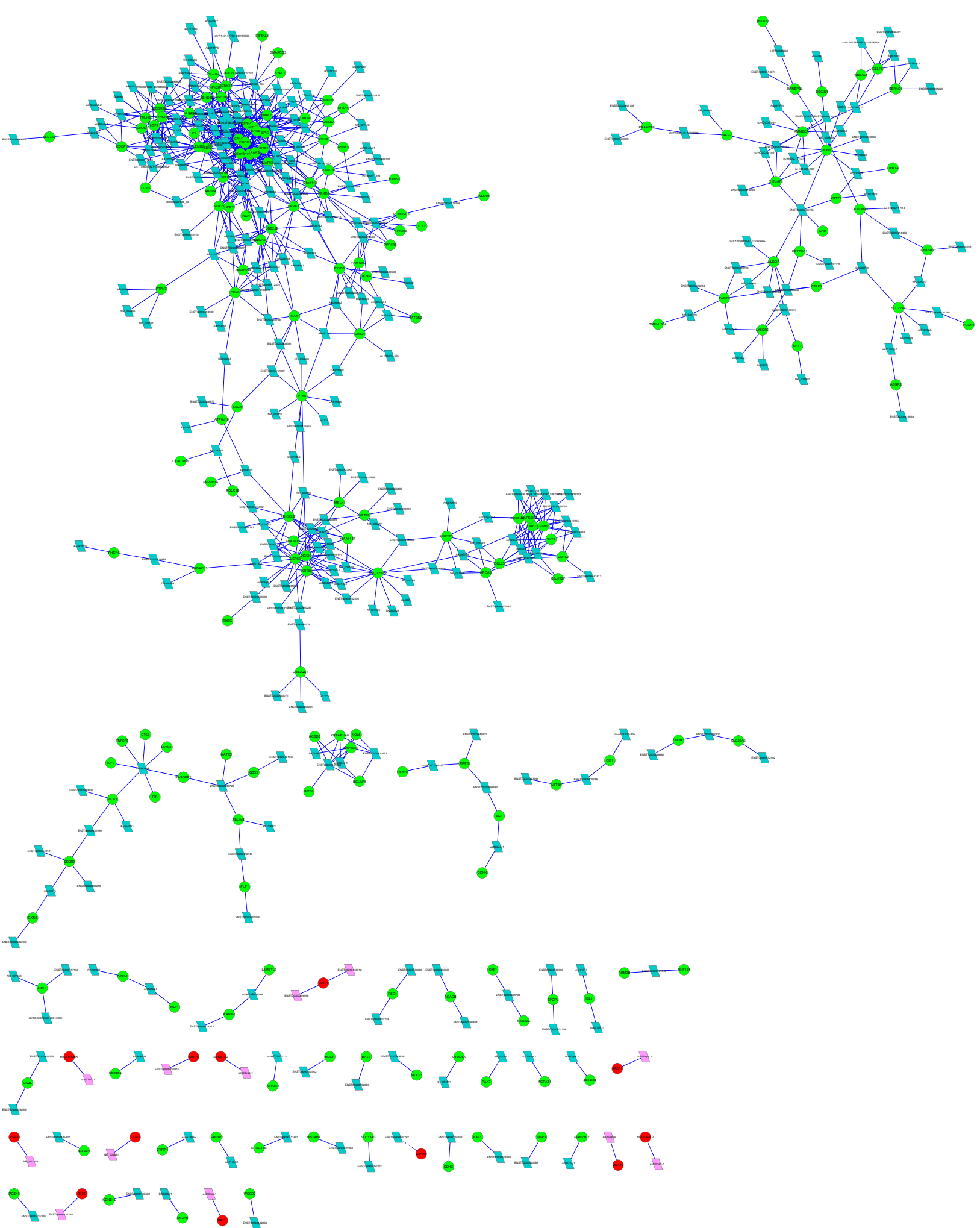

Supplement: Table S15 — CNC network. (PDF) [file pone.0042377.s015.pdf]
